# Immunohistochemical Study of Nrf2-Antioxidant Response Element as Indicator of Oxidative Stress Induced by Cadmium in Developing Rats

**DOI:** 10.1155/2015/570650

**Published:** 2015-05-25

**Authors:** Sergio Montes, Daniel Juárez-Rebollar, Concepción Nava-Ruíz, Aurora Sánchez-García, Yesica Heras-Romero, Camilo Rios, Marisela Méndez-Armenta

**Affiliations:** ^1^Departamento de Neuroquímica, Instituto Nacional de Neurología y Neurocirugía, Manuel Velasco Suárez, Insurgentes Sur 3877, La Fama, 14269 Tlalpan, DF, Mexico; ^2^Laboratorio de Neuropatología Experimental, Instituto Nacional de Neurología y Neurocirugía, Manuel Velasco Suárez, Insurgentes Sur 3877, La Fama, 14269 Tlalpan, DF, Mexico; ^3^Departamento de Bioterio, Experimental, Instituto Nacional de Neurología y Neurocirugía, Manuel Velasco Suárez, Insurgentes Sur 3877, La Fama, 14269 Tlalpan, DF, Mexico

## Abstract

In developing animals, Cadmium (Cd) induces toxicity to many organs including brain. Reactive oxygen species (ROS) are often implicated in Cd-inducedtoxicity and it has been clearly demonstrated that oxidative stress interferes with the expression of genes as well as transcriptional factors such as Nrf2-dependent Antioxidant Response Element (Nrf2-ARE). Cd-generated oxidative stress and elevated Nrf2 activity have been reported *in vitro* and *in situ* cells. In this study we evaluated the morphological changes and the expression pattern of Nrf2 and correlated them with the Cd concentrations in different ages of developing rats in heart, lung, kidney, liver, and brain. The Cd content in different organs of rats treated with the metal was increased in all ages assayed. Comparatively, lower Cd brain levels were found in rats intoxicated at the age of 12 days, then pups treated at 5, 10, or 15 days old, at the same metal dose. No evident changes, as a consequence of cadmium exposure, were evident in the morphological analysis in any of the ages assayed. However, Nrf2-ARE immunoreactivity was observed in 15-day-old rats exposed to Cd. Our results support that fully developed blood-brain barrier is an important protector against Cd entrance to brain and that Nrf2 increased expression is a part of protective mechanism against cadmium-induced toxicity.

## 1. Introduction

Cadmium, a heavy metal, is present in increasingly hazardous concentrations in soils, sediments, air, and water [1, 2]. A wide variety of health hazards in humans and experimental animals have previously been reported. Acute exposure to Cd house dust, smoking, and/or occupational exposure produces pulmonary edema and hemorrhage, followed by inflammation, scarring, pulmonary edema, and respiratory tract irritation; whereas chronic exposure to Cd often leads to renal dysfunction, the kidney is still regarded as critical organ for its accumulation and toxicity. The liver is the major target organ of toxicity following acute Cd poisoning, Cd-induced inflammation being an important mechanism for Cd-induced oxidative stress. Oxidative stress induced by Cd might be also one of the reasons for cardiovascular effects as low-density lipoprotein modification that is a key event in development of atherosclerosis [3–6]. Although the mechanisms of cadmium toxicity are poorly understood, one of the major mechanisms behind heavy metal toxicity has been attributed to oxidative stress [7–10].

Oxidative stress is generally defined as an imbalance that favors the production of Reactive Oxygen Species (ROS) and Reactive Nitrogen Species (RNS) over antioxidant defenses [9, 11, 12]. Cd is unable to directly generate free radicals; however, indirect mechanisms involve the generation of various radicals including the superoxide radical, hydroxyl radical, and nitric oxide [10, 13, 14]. The major consequence induced by Cd through oxidative stress is a ROS-mediated attack of double bonds in membrane lipids that results in increased lipid peroxidation (LPO) as well as interference with the endogenous antioxidant defenses in several organs and systems [5, 9, 15–17]. Acute or chronic intoxication of animals with Cd has shown increased activity of antioxidant defense system enzymes such as copper/zinc superoxide dismutase, catalase, glutathione reductase, and glutathione S-transferase [17–20]. ROS are predominantly implicated and it has been clearly demonstrated that oxidative stress interferes with the expression of genes as well as several transcriptional factors such as metal regulatory transcription factor 1 (MTF1), AP-1 upstream stimulator factor (USF), nuclear factor-B (NF-*κ*B), and NF-E2-related factor (Nrf2). ROS may function as secondary messengers that deregulate gene expression and induce cell transformation when cells are exposed to Cd [6, 21–23]. Nrf2-dependent Antioxidant Response Element (Nrf2-ARE) is a basic leucine zipper transcription factor that binds to antioxidant responsive element (ARE); this factor is a critical regulator of effective cellular response, and its function confers cellular protection to oxidative stress [24]. The nuclear accumulation of Nrf2 is an essential signaling step for its function as a transcription factor [25, 26]. In many cell types the exposure to Cd generated oxidative stress; this phenomenon, in turn, has been associated with elevated Nrf2 activity that results in increased expression of some antioxidant enzymes [27, 28].

One important factor linked to brain Cd toxicity is age; experimental evidence has shown that in adult rats only small amounts of Cd reach brain, whereas in the newborn and developing animals Cd readily reaches brain [29–31]. This effect might be due to differences in the brain-blood barrier (BBB) maturation; therefore, in the present study, developing rats were intoxicated with cadmium at different ages, before BBB consolidation (5-, 10-, and 15-day-old) and after BBB rodent strengthens (21-day-old) to confirm that BBB consolidation limits Cd entrance to brain. In addition, we were interested to know the distribution of Cd after exposure in neonates, especially to grossly explore the kinetics of Cd in some brain areas and some other tissues; thus newborn rats were injected with cadmium and the content of metal was determined along time. We also look for morphological damage in brain from Cd-intoxicated pups and the expression of a transcriptional factor associated with oxidative stress defensive response.

## 2. Material and Methods

### 2.1. Animals

Experiments were performed using male and female Wistar rats; NIH bred in-house strain newborn or 1, 5, 10, 15 and 21 days old. Animals were maintained in standard conditions (light period 7:00 AM–7:00 PM, at 22–24°C, and humidity was 40%) during all the experimental period. The newborn rats were allowed to remain with their dams throughout the experiment. All animals were fed on a standard chow diet (Purine Chow) and given water* ad libitum*. Rats were individually housed in polycarbonate cages. Animals were handled according to the National Institutes of Health (USA) Guidelines for the Care and Use of Laboratory Animals, and this protocol was approved by the Bioethics Committee of the National Institute of Neurology and Neurosurgery of Mexico.

### 2.2. Treatment

#### 2.2.1. Time-Course of Cadmium Tissue Content in Newborn Rats

Newborns were randomly assigned to different experimental groups and were injected i.p. with cadmium chloride (1 mg Cd/kg) dissolved in saline (0.9% NaCl w : v); the control group received saline solution (0.9% NaCl w : v); similar doses have been used previously by us [3, 16, 32]. Rats were sacrificed 2, 24, 48, and 72 hours after single doses of Cd or saline administration to determine Cd content in lung, liver, kidney, and heart.

On the other hand, to find out the participation of age into Cd tissue distribution, rats aged 1, 5, 10, 15, and 21 days were injected at the appropriate times with cadmium chloride (1 mg Cd/kg) and then sacrificed 24 h after the metal injection to determine tissue cadmium content. From every experimental group, four animals were randomly selected for histological examination, and six animals were used for cadmium content analysis.

### 2.3. Cadmium Determinations

Cadmium content in tissues was analyzed by graphite furnace atomic absorption spectrophotometry (GFAAS) (PerkinElmer AAnalyst 600), according to the technique described by Christian, 1969 [33] and using the conditions described by Eller and Haartz (1977) [34]. The brain regions were dissected out in parietal cortex (Cx), striatum (St), hippocampus (Hp), and cerebellum (Ce) [35]. Tissue samples were digested in 1 mL of concentrated HNO_3_. Calibration curves were constructed using an aqueous Cd reference standard (GFAA mixed standard, PerkinElmer). As biological-matrix external standard, we used nitric acid-digested bovine liver (NIST 1577b) standard reference material, and this certified standard was analyzed in every analytical session; values obtained from that analysis were 95–105% from those reported by manufacturer. Cd content was expressed as *μ*g of Cd/g wet tissue. All glassware was cleaned by soaking in 3% nitric acid and rinsing several times in deionized water. The limit of quantification for Cd analysis was 0.01 *μ*g/g wet tissue.

### 2.4. Histopathological and Immunohistochemical Study

For histological examination, animals were anesthetized with pentobarbital (40 mg/Kg) and then perfused with cold saline solution followed by 10% formaldehyde at 4°C and the heart, lung, kidney, liver, and brain were rapidly removed. Tissue samples were processed separately in a Histokinette 200 apparatus (Reichert Jung). Sections were paraffin-embedded, cut into 5 *μ*m slices and hematoxylin-eosin stained [36], and examined under light microscope (AXIOLAB, Carl Zeiss) to verify tissue integrity.

On the other hand, sections were incubated with monoclonal antibody against Nrf2 1 : 100 (Nrf2 Santa Cruz Laboratory). Sections were then incubated with a Biotin-conjugated secondary antibody and Streptavidin-Enzyme Conjugate (LSAB System HRP, BIOCARE). The immune reaction resulted in the oxidation of the 3,3′-diaminobenzidine by peroxidase (Liquid DAB, DAKO Carpinteria, CA) into an insoluble brown precipitate. The reaction sites were visualized as a brown staining. Counterstaining with hematoxylin was performed after immunostaining.

### 2.5. Statistical Analysis

Statistical analyses were performed by using SPSS V.19.0 statistical software package. Data are presented as mean ± standard error of the mean. Normal distribution and homogeneity of data were confirmed before one-way ANOVA, followed by Tukey's test. A level of *p* < 0.05 was considered to be statistically significant.

## 3. Results

### 3.1. Time-Course of Cadmium Tissue Content in Newborn Rats

**Figure 1:**
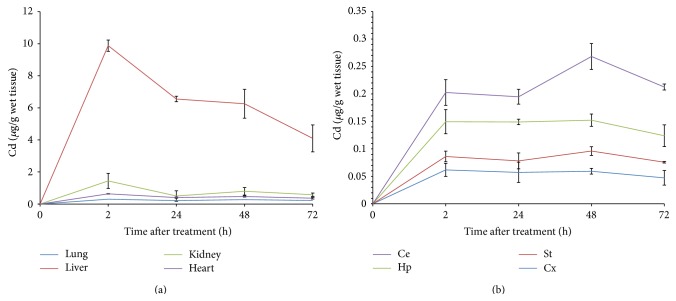
(a) Time-course of cadmium tissue content in newborn rats and (b) time-course of cadmium content in brain regions from newborn rats. Cadmium (1 mg/kg) was administered i.p. to newborn animals and cadmium was measured in the tissues at different times after injection. Values represent mean ± SEM from six animals per group expressed as Cd *μ*g/g of wet tissue.

The time-course of Cd content in lung, heart, liver, and kidney of newborn rats at different times after single dose Cd administration is observed in Figure 1(a). Among the organs explored, liver accumulated far higher Cd than the other organs; we found that, two hours after injection of metal, the content of Cd in the liver was 9.88 *μ*g/g, followed by elimination phase decreasing to 4 *μ*g/g 72 hours after metal injection. Kidney also showed a peak at two hours but the concentration found was one-tenth from liver. Heart and lung showed comparatively no appreciable content of the metal.

Cd distribution in the different brain regions assayed is shown in Figure 1(b). Cerebellum was the brain area with the highest Cd content followed by hippocampus, striatum, and cortex. The distribution in cerebellum showed a peak 48 h after acute Cd treatment. At the times assayed, we did not observe the Cd elimination phase from brain areas.

### 3.2. Cadmium Content after Injection of Metal to Rats at Different Ages

**Figure 2:**
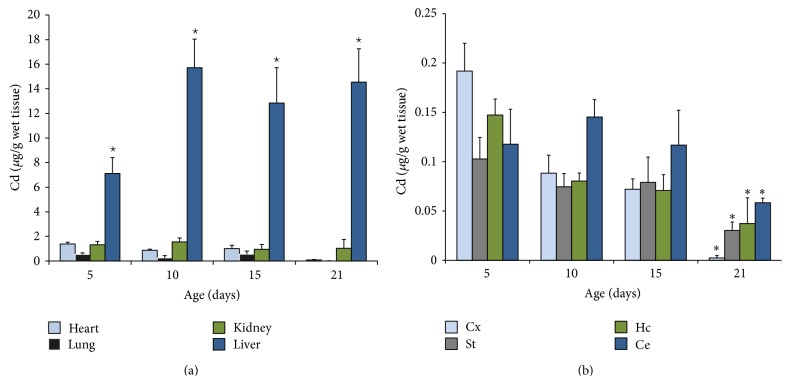
(a) Cadmium tissue concentrations from rats pups treated with cadmium at different ages and (b) cadmium brain regions from the same animals. Rats were injected i.p. with cadmium (1 mg/kg) and 24 h later they were sacrificed to determined cadmium levels. Values represent mean ± SEM from six animals per group expressed as Cd *μ*g/g of wet tissue.

The Cd content in different organs in function of age is shown in Figure 2(a). The content of Cd in heart, lung, liver, and kidney of animals was analyzed 24 h after Cd injection to 5-, 10-, 15-, and 21-day-old animals. We found that the lung and the heart slightly increased the content of the metal at all ages analyzed; no statistical differences were found in Cd distribution for these organs. The kidney showed an important increase in Cd levels at 5- (1.32 ± 0.26 *μ*g/g wet tissue), 10- (1.55 ± 0.31 *μ*g/g), 15- (0.95 ± 0.3 *μ*g/g), and 21-day-old (1.04 ± 0.07 *μ*g/g) rats. In contrast, the highest Cd concentration observed in this study was in the liver at all ages analyzed.

Figure 2(b) shows the Cd content in the four brain regions assayed. We observed that the metal content was increased in all brain regions, and this effect was evident in those 5-, 10-, and 15-day-old rats. In contrast, in 21-day-old rats the Cd content was comparatively and statistically (*p* < 0.05) lower than those other ages assayed. No differences were observed among brain regions.

### 3.3. Histopathological Examination

**Figure 3:**
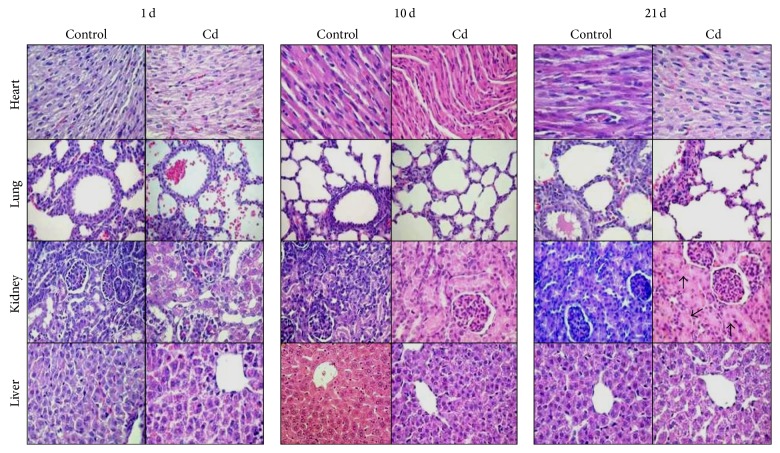
Histopathological analysis in cardiac muscle lung, kidney, and liver tissue sections obtained from 1-, 10-, and 21-day-old Cd-treated pup rats. Swollen renal tubular epithelial cells with displacement of nucleous and a pale appearance were observed (↑). Hematoxylin-eosin staining, magnification 400x.

**Figure 4:**
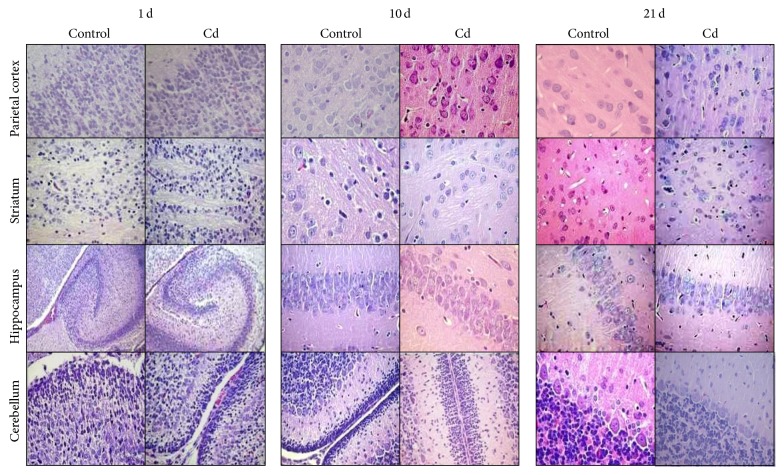
Histopathological analysis in brain regions. Parietal cortex, striatum, hippocampus and cerebellum sections obtained from 1, 10 and 21 days-old rats. Hematoxylin-eosin staining, magnification 400x.

**Figure 5:**
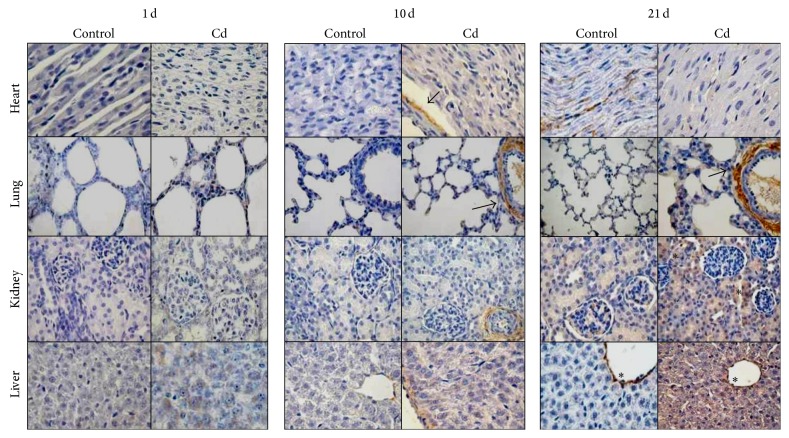
Immunohistochemical study of Nrf2 in cardiac muscle, lung, kidney, and liver tissue sections obtained from 1-, 10-, and 21-day-old rats. A few Nrf2 positive cells are seen in the control group at the age of 21 days. Tissues increased Nrf2 immunoreactivity in vascular smooth muscle of arteriole (↑), tubular proximal cells of kidney (∗), hepatocytes, and central vein of liver (∗) of rats in the experimental group. Magnification 400x.

**Figure 6:**
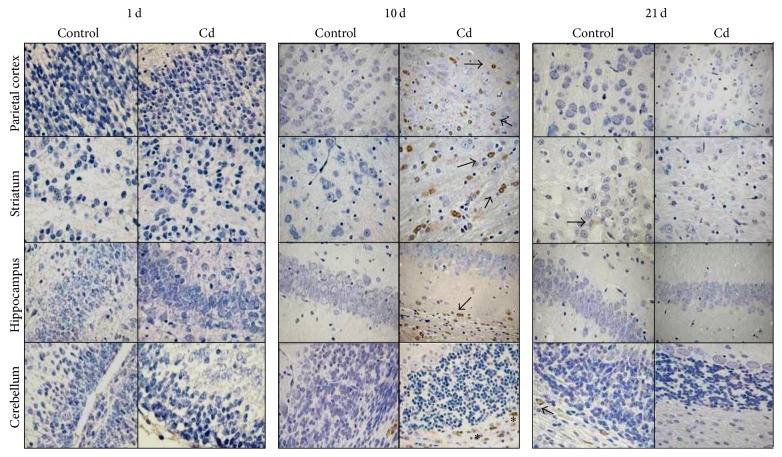
Immunohistochemical study of Nrf2 in brain regions. Parietal cortex, striatum, hippocampus, and cerebellum sections obtained from 1-, 10-, and 21-day-old rats. Scarce Nrf2 positive cells are seen in the control group at 21 days old (↑). Increased Nrf2 immunoreactivity was observed in cerebellum oligodendrocytes (∗) and glial cells (↑) of the other brain regions in the experimental group. Magnification 400x.

In Figures 3, 4, 5, and 6 we compared the histological structure of different tissues from control and Cd-treated animals at different ages using hematoxylin-eosin and immunohistochemistry for Nrf2 as oxidative stress response marker. Histological examination of Cd-treated rats revealed minimal morphological changes as compared to those of controls in the heart, lung, kidney, liver, and brain of 5-, 10-, and 21-day-old rats. In Figure 3 the cardiac muscle of control group has shown fibers grouped in bounds, connective tissue with capillaries between the cardiac fibers in the control and treated groups; no lesions were observed in the Cd-treated groups (Figure 3). Representative lung sections obtained from controls and Cd-exposed animals in the light microscope are also shown in Figure 3. No lesions were evident since; conserved alveolar basement membranes and vascular endothelial cells, as well as bronchioles with alveolar ducts, were observed in the experimental and control groups.

The morphological findings from the current study revealed slight changes in the kidney of 21-day-old Cd-exposed rats. In the control group normal structure of glomeruli, the proximal tubular epithelium, tubules, and basement membrane showed normal appearance (Figure 3); in contrast, swollen renal tubular epithelial cells with displacement of nucleolus and pale appearance were observed in the Cd-exposed rats (Figure 3). Liver preparations from controls showed normal hepatic architecture with presence of a central vein surrounded by normal radiating hepatic cords with normal sinusoids in-between (Figure 3). Liver from Cd-intoxicated rats showed normal hepatic lobules, consisting of a central vein surrounded by radiating hepatocytes plates with few leukocytic infiltrations (Figure 3).

Figure 4 shows brain regions slides under light microscope examination. In control rats, they appear with normal histological structure as expected, that is, the cytoplasm of the neuronal cell body with Nissl substance, pyramidal, and Purkinje cells with no apparent alterations (Figure 4). Rats exposed to Cd showed minimal alteration of neuronal cells and neuropil in brain regions analyzed, with slight interstitial edema and few pyknotic cells in Cx and Ce in 5-day-old (data not shown) and 15-day-old rats exposed to Cd (Figure 4).

### 3.4. Nrf2 Immunohistochemical Study

We performed immunohistochemistry to identify the Nrf2 in several organs from Cd-exposed rats. Immunohistochemical examination revealed negative immunostaining for Nrf2 in heart, lung, and kidney sections of control groups from 1- and 5-day-old rats, whereas slight immunoreactivity was observed in liver of 1- and 5-day-old rats both exposed to Cd (Figure 5). In contrast, compared to the controls, increased Nrf2 immunostaining was detected in the vascular smooth muscle of arteriole in heart and lung; basement membrane and tubular proximal cells of kidney, hepatocytes and central vein of liver (Figure 5) from 21 days-old Cd exposed-rats also shown similar immunostaining.

We found scarce immunoreactivity for Nrf2 in brain regions on both 1- and 5-day-old rat groups. Increased immunoreactivity was observed in oligodendrocytes from Ce brain region in 10-day-old rats (Figure 6). However, slight Nrf2 immunostaining was detected in the glial cells of all brain regions analyzed of rats treated with Cd at 21 days old (Figure 6).

## 4. Discussion

In this study, we investigated the distribution of Cd in several organs of the developing rat. Our results showed that the administration of Cd to the developing newborn rats was able to increase the concentrations of this metal in some organs during early postnatal development. High content of Cd, from 2 h after metal injection, suggested a fast distribution to liver and kidney. In brain we also observed Cd in the different brain areas, from 2 h and maintained along 72 h after metal injection. In cerebellum, which also was the brain area with the highest Cd content from brain areas assayed, it showed a peak 48 h after Cd treatment. The presence of Cd in the newborn rats in short periods suggests a fast distribution to organs, including brain.

The ability of Cd to accumulate is dependent on its binding to ligands present inside the cell; Cd is widely distributed in the body with the major body burden located in the liver and kidney. Cd content builds up with age and the rate of accumulation is significantly higher during fast growing phases of animals [37]; similar results were also report by Wong and Klaassen [38, 39] and Manca et al., [15] who found that the retention of Cd was higher in the liver, kidney, and lung in the newborn rat than in the adult rat after cadmium exposure. Our results (Figures 1, 2, and 3) are in agreement with those reports. In contrast and of particular importance, several studies have suggested that age of animals is very important to make them susceptible to the neurotoxic effects of Cd due to the BBB immaturity [5, 29, 32]. According to those reports, and the results from the present study, the evidence suggests that developing rats are very susceptible to increase the Cd concentration in the brain regions assayed (Ce < St < Hc). When we compared the Cd content from Cd-treated rats with different ages we found that 21-day-old rats accumulated comparatively less metal in brain than younger pups (Figure 4) confirming that the presence of the mature BBB enables protection against metal brain accumulation; therefore Cd is more toxic to newborn and young rats than to adult rats.

Oxidative stress is considered the major event underlying Cd toxicity, being able to produce tissue damage. However, in our histopathological examination, no apparent changes were observed after Cd treatment in the morphology of heart, lung, kidney, liver, or brain. Only minor renal damage was observed in 21-day-old rats (Figures 3 and 4). This effect was not observed in younger pups, probably because the time to evaluate damage after Cd treatment was short. In this regard, Cd is absorbed and distributed to different tissues and its toxicity depends, mainly, on the chemical form and the time of exposure [40]. Several studies have demonstrated that histopathological damage, as well as renal and liver necrosis, myocardial damage, vascular edema, testicular hemorrhage, pulmonary fibrosis, neuromyopathy, and brain damage among others, occurs mainly upon chronic Cd exposure, both in humans and in experimental animals [5].

Cd-induced ROS production is associated with increased oxidative stress and in turn with transcription factors such as Nrf2 [21]. Nrf2-ARE has been considered to be the key regulator of oxidative stress by modulating the expression of antioxidant and detoxification enzymes. The activation of Nrf2 by Cd involved the stabilization of the Nrf2 protein, increased formation of Nrf2/Keap1 complex in the cytoplasm, translocation of the complex to the nucleus, and subsequently disruption of the complex [41]. Several studies have reported the changes in the transcriptional regulation of Nrf2 by Cd in several cell lines [28] and mouse embryonic fibroblasts [41]. The overexpression of Nrf2 significantly reduced the apoptotic cell death in kidney proximal tubule in cells treated with chromium [42] and in kidney cells exposed to cadmium [27]. These results suggest that in kidney cells the activation of Nrf2 is an adaptive intracellular response to Cd-induced oxidative stress and that Nrf2 is protective against Cd-induced apoptosis [23]. In agreement with those reports, here we show that Nrf2 was localized in endothelial, smooth muscle, and glial cells from Cd-treated rats. The assay also showed negative Nrf2 immunoreactivity in analyzed tissues from both control and Cd-treated animals at the age of 1 and 5 days old (Figures 5 and 6). In contrast, Nrf2 immunoreactivity was increased in all tissues from 10- and 15-day-old metal-treated rats, whereas limited Nrf2 immunoreactivity was observed in brain regions from 21-day-old Cd-treated rats, a phenomenon consistently related with the limited entrance of Cd to the brain at the same age (Figure 6). These results are consistent with the evidence from Chen and Shaikh [27] showing that Cd exposure increases the expression of Nrf2 probably as a compensatory mechanism to oxidative stress to counteract the toxic effects caused by Cd.

In summary, this study shows that the exposure to Cd in developing rats induce the accumulation of this metal in several tissues including the central nervous system. However, the Cd entrance to brain is limited by a developed blood-brain barrier. Immunohistochemical staining demonstrated that high level expression of Nrf2 was present in several cellular types including glial cells and that Nrf2 immunoreactivity was increased in rats exposed to Cd. Our results support that Nrf2 increased expression is an intracellular response against Cd toxicity.
